# Content and communication: How can peer review provide helpful feedback about the writing?

**DOI:** 10.1186/1471-2288-8-3

**Published:** 2008-01-31

**Authors:** Karen Shashok

**Affiliations:** 1 Translator and Editorial consultant, Compositor Ruiz Aznar 12, 2-A 18008 GRANADA, Spain

## Abstract

**Background:**

Peer review is assumed to improve the quality of research reports as tools for scientific communication, yet strong evidence that this outcome is obtained consistently has been elusive. Failure to distinguish between aspects of discipline-specific content and aspects of the writing or use of language may account for some deficiencies in current peer review processes.

**Discussion:**

The process and outcomes of peer review may be analyzed along two dimensions: 1) identifying scientific or technical content that is useful to other researchers (i.e., its "screening" function), and 2) improving research articles as tools for communication (i.e., its "improving" function). However, editors and reviewers do not always distinguish clearly between content criteria and writing criteria. When peer reviewers confuse content and writing, their feedback can be misunderstood by authors, who may modify texts in ways that do not make the readers' job easier. When researchers in peer review confuse the two dimensions, this can lead to content validity problems that foil attempts to define informative variables and outcome measures, and thus prevent clear trends from emerging. Research on writing, revising and editing suggests some reasons why peer review is not always as effective as it might be in improving what is written.

**Summary:**

Peer review could be improved if stakeholders were more aware of variations in gatekeepers' (reviewers' and editors') ability to provide feedback about the content or the writing. Gatekeepers, academic literacy researchers, and wordface professionals (author's editors, medical writers and translators) could work together to discover the types of feedback authors find most useful. I offer suggestions to help editologists design better studies of peer review which could make the process an even stronger tool for manuscript improvement than it is now.

## Background

Editorial interventions by gatekeepers (reviewers and editors) of scientific, technical and medical (STM) communication can be classified into two types: those meant to help make the discipline-specific content meet the journal's or publisher's editorial requirements (their "screening" function), and those aimed at making the text more convincing as a written communication (their "improving" function). This article examines elements of the peer review process to see whether the features reviewers are asked to evaluate can be distinguished as relevant to either the scientific content or the writing. A provisional classification of editorial policies and guidelines for reviewers suggests that although these two types of feedback are often requested, gatekeepers may fail to fully appreciate the difference between the two. Research on peer review has also tended to confuse the two dimensions–a methodological shortcoming that may explain why much peer review research in biomedicine has yielded so little unequivocal evidence that the process improves the quality of what is published.

To document the evidence that peer review feedback about language and writing may be less useful than gatekeepers assume, I report observations by author's editors (language and writing specialists who help authors write and revise their material more effectively) [1] regarding the quality of the feedback authors receive from gatekeepers. I also review some of the descriptive data about the objectives of peer review obtained from editorial guidelines for authors and reviewers. Research by language and communication specialists in academic writing that has implications for peer review is also examined.

I propose a simple classification system intended to help gatekeepers distinguish which of the two quality dimensions–specialized content vs. the use of language and writing–evaluation criteria and comments provided by reviewers pertain to. To conclude, I suggest that pooling knowledge from three specialist communities–journal editors, researchers in language and communication, and wordface professionals such as author's editors, medical writers and translators–would lead to improvements in peer review practice and better research on this complex process.

## Discussion

### What is peer review assumed to accomplish?

Peer review is considered 1) a screening instrument which lets some material through the gates but refuses entry to other submittals, and 2) an editing instrument that turns articles allowed through the gates into better-written or better-edited texts. Experts in peer review have suggested that "the two principal functions of peer review" are "filtering out incorrect or inadequate work and improving the accuracy and clarity of published reports." [2]. These functions have been further categorized as (1) "selecting submissions for publication" and "rejecting those with irrelevant, trivial, weak, misleading, or potentially harmful content," and "(2) improving the clarity, transparency, accuracy, and utility of the selected submissions." [3] Distinguishing between the ability to evaluate the scientific content (i.e., the "selection" "gatekeeping," "screening" or "deciding what gets published" functions of peer review) and the ability to provide effective feedback on the content, writing or language (i.e., the "improving what gets accepted" function of peer review) would help make explicit which skills make peer reviewers useful to editors and authors. This is important because the ability of peer review to perform the "improving" function effectively has been questioned not only by wordface professionals [4] but by researchers in peer review [5].

Some editors [6] have found that even careful, prospective research cannot reliably identify characteristics of good reviewers, ways to train reviewers to become better, or characteristics that contribute to good reviewing skills. A recent editorial in *Nature *also recognized the problem with peer review quality:

What right has [an author] to expect a high quality of peer review? What training is being given in his or her own lab to ensure that the next generation understands how to do a good job of critically appraising others' work? And as the pressures on researchers grow–bureaucracy from institutions and funding agencies, incentives to apply the outcomes of research–the very motivation to do a conscientious job of peer review is itself under pressure [7].

Many editors seem to be unaware that the ability to provide helpful feedback on different quality dimensions requires skills which cannot be assumed to be "standard equipment" in all potential reviewers. A hypothesis worth considering is that discipline-specific content is more likely to be judged objectively because this is where gatekeepers' expertise is greatest. In contrast, language and writing features are more likely to be judged subjectively because gatekeepers' expertise in this dimension varies widely. The latter is probably influenced by individual characteristics such as the reader's native language and culture, and personal preference for language and writing style [8]. As a result, feedback about the language and writing may be less likely to help authors improve their manuscripts than feedback about the specialized content.

### Evidence of unhelpful feedback about the language and writing

Author's editors and translators who help authors interpret reviewers' feedback frequently observe that reviewers are quick to complain about "the English." Although reviewers sometimes correctly identify problems with technical language or first-language interference, they often claim that a manuscript requires "substantial review and editing by a native English speaker" when in fact they may be reacting to usage or argumentation that is appropriate but different from their preferred style. Below I list some of the changes made or requested by gatekeepers that can make the text harder instead of easier to understand.

1. Edits to improve "good scientific English style": the corrections can introduce unfortunate word choices, jargon, undefined or unneeded abbreviations, and other technical editing errors.

2. Changes in terminology and nomenclature: the reviewer's knowledge may not be up-to-date.

3. Corrections in grammar and syntax: reviewers may overestimate their proficiency in written English.

4. Changes in organization: reviewers may request changes that disrupt the logical flow of ideas.

5. Changes in argumentation and rhetoric: sometimes "non-standard" rhetorical strategies used by authors are more appropriate than the type of writing the reviewer prefers.

Wordface professionals often agree with researchers who feel reviewers have provided contradictory feedback about the writing or complained about "the English" even when native speakers of English wrote, translated or revised the material. Table 1 shows the frequency with which feedback about the English or the writing was considered unhelpful by a sample of experienced STM translators, author's editors and medical writers.

**Table 1:** Native-English-speaking author's editors' perceptions of the usefulness of feedback from journal gatekeepers about the language. Questionnaire survey, October 2007. N = 25, response rate 40%.

Total number of manuscripts handled	Percentage of manuscripts with complaints about the language or writing	Percentage of correct comments or changes	Percentage of incorrect comments or changes
20	60	40	60
50	10	70	30
100	10–20	80–90	10–20
200	5	75	25
300	5–10	0	100
300	5	50	25
>300	<10	30	10
1200	2–3	75	25
3000	30	50	50
3000	<10	Occasional	0
Several thousand	Very common		Often^a^
Range	5–60	0–90	0–100
Mean	15.5^b^	46.2^c^	32.3^d^
Mode	10	50–75	25

^a ^Data from this respondent were not included in the descriptive statistical analysis.

^b ^<10 was entered as 10; the value midway between the two extremes was entered when a range of values was given.

^c ^Occasional was entered as 50, the value midway between the two extremes was entered when a range of values was given.

^d ^The value midway between the two extremes was entered when a range of values was given.

Although consensus between reviewers is not necessarily one of the aims of peer review, contradictory feedback about the writing is unhelpful if not accompanied by guidance from the editor. The unhelpful comments made by some reviewers may reflect their tendency to consider their role as "one of policing rather than identification of work that is interesting and worth publishing." [9] As gatekeepers, some reviewers may assume it is more important to find reasons to reject a submittal than to help make worthy but imperfectly polished manuscripts better. As busy professionals with limited time to spare for non-remunerated but demanding work, reviewers may be more highly motivated to find a few fatal flaws than to undertake the more time-consuming task of providing constructive feedback.

Although many additions reviewers suggest do improve research articles, an undesirable outcome of peer review is the introduction of changes that the authors know to be wrong but which are added "to conform to the referee's comments." [9] Reviewers' comments that force authors to rewrite a paper "in ways that sometimes do not support, but rather weaken" their arguments have been a concern in social science disciplines for decades [10]. Researchers I have worked with have, at the reviewer's behest, added unnecessary citations and even whole paragraphs which had the unfortunate side effect of disrupting the logical flow of ideas. As a result published articles may be less coherent, less persuasive, and less attractive to readers than they might have been if the reviewers had shown more flexibility and asked themselves whether their suggested changes actually improved the text.

### Decline in editorial tolerance for writing that departs from readers' expectations

Many authors do not have ready access to professional editorial help – a problem with the potential to worsen the North-South and West-East information imbalance [11,12]. Moreover, reviewers and editors may no longer be as willing or able as they were before to provide extensive help with the writing or language [13]. Programs such as AuthorAID will attempt to palliate geographical imbalance in access to high-quality author editing and language help [11].

Meanwhile, journals in some disciplines seem to be abandoning manuscript editing, a trend which seems to parallel a similar decline in editorial tolerance for imperfect English. To study the trend among STM journals to dispense with editing, I compared policies at four large commercial publishers: Springer, Elsevier, Wiley and Blackwell. (The latter two publishers merged in February 2007). Current policies, discussed here, [See additional file 1: Publishers' language policies] reflect a range of positions from an appreciation of authors' difficulties in writing well to explicit statements that the publisher is not prepared to edit accepted manuscripts.

Although trends differ between disciplines, recent years have seen a decrease in the number of journals that are willing or able to undertake high-quality editing. For example, in 1993 Jill Whitehouse, then Executive Editor of *Physiotherapy*, published an article titled "Readability and clarity" in which she described "the responsibilities of reviewers of articles in helping authors improve their writing style." Reviewers for this journal were expected to provide feedback on both the content and the "style," defined by this editor as features that enhanced "clarity of communication and elegance." [14]

Currently the journal, published by Elsevier, offers sparse advice about the standard of writing or language authors are expected to meet: "Please write your text in good English (American or British usage is accepted, but not a mixture of these)." [15] There is no longer any indication that reviewers or editors consider it their job to attend to "style".

Debate among editors on the WAME listserve in late 1999 reflected the change in attitude toward the effect of language and writing on a manuscript's chances of acceptance. Robin Fox wondered whether "pragmatism will prevail over fairness," and editors debated what could be done to ensure that the quality of the writing was as good as the quality of the content [16]. Some editors felt the language burden created an uneven playing field that posed additional obstacles to publication for researchers whose first language is not English. Some said they were glad to spend extra time on manuscripts with language or writing problems. However, a few editors admitted that because of practical considerations it might be necessary to reject manuscripts that reported good work if they needed too much editing (i.e., more editing than the editor or publisher could afford to provide).

The latest edition of the American Medical Association (AMA) style manual offers no advice on writing or text revision but contains an abundance of rules on specific points of grammar, usage and technical style [17]. Although it is considered a de facto standard for medical publishing in English (at least in the USA), the AMA manual lacks advice on the type of writing gatekeepers at biomedical journals are likely to find acceptable. It does, however, note that poor writing is considered a legitimate reason to reject a manuscript (p. 265).

To compare policies across disciplines I also looked at how the style manuals of the American Psychological Association and American Chemical Society [See additional file 2, American Psychological Association and American Chemical Society language policies] handle peer review of the language and writing.

My own experience with manuscripts published in different journals since the mid-1980s suggests that in general, only the biggest, wealthiest, highest-impact-factor journals continue to provide good copyediting as part of their added value services. Current practices are changing and differ between journals and between publishers, so reviewers may feel confused as to what they are expected to comment on. As a result they may assume that they should attempt to improve the writing or language even if (or perhaps precisely because) it is no longer the journal's or publisher's policy to provide this service.

### Application of a two-category coding system for content analysis

Analyzing the guidelines for reviewers according to the two quality dimensions suggested here–specialized content and writing–will show which criteria are likely to be evaluated more objectively and which are likely to be evaluated more subjectively. The criteria used to judge the specialized content should help answer the question, "Does the manuscript report questions, findings and ideas that readers ought to know about?" The criteria used to judge the writing should help answer the question, "Will readers understand well enough what the authors are trying to say?"

Coding advice reliably as pertaining to either the content or the writing requires a taxonomy of features that can be identified easily and reproducibly. Table 2 shows a tentative list of words and phrases that label instructions or comments as relating to one dimension or the other.

**Table 2:** Markers of content-related and writing-related information in guidelines and feedback intended for authors and reviewers

Content-related information	Writing- or language-related information
Verbs denoting intellectual processes: Interpret, analyze, discuss, assume, claim, synthesize, conclude	Verbs denoting communication processes: Say, write, explain, clarify, mention, mean, claim, argue, maintain, suggest

Nouns denoting understanding of the content: Interpretation, analysis, discussion, conclusion, assumptions, significance, reasoning, context, evidence, information, data, hypothesis, experimental design	Nouns denoting elements of the text: Word, phrase, sentence, paragraph, section, text, manuscript, article, typo, misspelling, grammar, style, writing, explanation, flow, message, organization, readability, English, writing, language

Judgments denoting readers' reactions to the content: Uninteresting/Interesting, unoriginal/original, innovative, valid, relevant, intriguing, trivial, superficial	Judgments denoting readers' reactions to the text as a written communication: Hard/Easy to follow, well/poorly written, understandable, unclear/clear, confusing, unconvincing/convincing, well/inadequately documented

As a preliminary test of the usefulness of using just two categories to classify the content, I analyzed different types of texts that contain advice for authors or reviewers. The results of this exercise are reported here. [See additional file 3: Test of the 2-category coding system]

These preliminary quantitative analyses suggest that the 2-category system is applicable, but replication by many more raters is needed with a large sample of instructions to reviewers, reviewers' reports and instructions to authors.

### Other content analysis studies of quality criteria

As shown in an analysis of 35 sets of instructions to authors by Schriger at al. [18], there are unresolved issues with content validity. Study 2 in this article counted the frequencies of words pertaining to 18 different categories grouped into 4 major classes. Only 5 journals devoted more than 10% of the words to scientific content. Although differences in the classification method and the type of document analyzed make comparisons problematic, their low figures for content-related criteria contrast with my preliminary finding that 71% of the criteria reviewers were asked to consider pertained to the content (Table 3 in reference 18). None of the 18 categories considered by Schriger and colleagues were related specifically to the quality of the language or writing. However, their "scientific content" class included 3 categories for "content or style," "methodology or statistics" and "general content." This last category included instructions about format and style along with information that could not be assigned to any of the other 17 categories.

So the reason for the large difference in content-related criteria between the classification by Schriger and colleagues and the 2-category system proposed here is probably because what Schriger and colleagues called "content" in their analysis comprised a mixture of advice on format, style and reporting, and so cannot be compared to "content" considered here as hypothesis, experimental design, data and analysis.

At issue, however, is not the magnitude of the difference in the proportion of comments considered to pertain to content. The methodological issue here is that the two analyses cannot be compared because of the differences in how content-related comments are defined and classified by different authors. Difficulties in defining text-based variables for content analysis were noted in a similar study that compared comments to authors provided by methodology and regular reviewers [19]. The methodological pitfalls of content analysis aimed at "deciding which comments refer to which text features" were also pointed out by Belcher in a study of reviewer feedback to authors whose first language was not English [20].

Other categories in addition to content and writing hold potential to shed light on the peer review process. One potentially useful category is "reporting" since the damage weak reporting does to scientific communication is now clear [21]. The reason so much weak reporting reaches print is because peer review fails to detect and correct faults, so training gatekeepers in how to identify problems with study design, methodology, statistical analysis and data reporting is one way to make peer review more effective.

A recent paper in *BMC Medical Research Methodology *[22] classified comments about manuscripts as pertaining to science (i.e., content), journalism or writing. The JAMA study used a third category (journalism) because this leading journal, like other high-impact publications, considers many non-content-related factors in its peer review decisions [23]. Most journals, however, could probably obtain useful information with content and writing as the sole classification criteria.

### Insights into peer review by language and writing specialists and wordface professionals

Academic research in communication disciplines is helping to bring into focus some of the issues peer review research by gatekeepers has so far failed to consider. Some of this research is reviewed here. [See additional file 4: Academic research] Joy Burrough-Boenisch, a translator, author's editor and specialist in language for specific purposes, has worked with researchers from different linguistic, cultural and academic backgrounds to investigate readers' expectations for academic texts across a range of disciplines and native languages [24]. Her groundbreaking multidisciplinary research yielded findings that gatekeepers interested in serving their readers well might find stimulating. The findings, summarized here, [See additional file 5: Wordface research] support the notion that advice on "the writing" offered by scientific peers may be less helpful to authors than advice offered by professional editors or other communication professionals.

The reasons for this are not hard to grasp when the skills of discipline specialists and communication specialists are compared. Text revisers such as translators, language editors and copyeditors tend to make changes to improve readability, at least on a sentence or paragraph level. But if they are not subject experts, language professionals or copyeditors may miss deficiencies in the logic and argumentation because they do not grasp the scientific content. In contrast, peer reviewers (ideally) focus on the validity of the actual scientific content and reporting, and flag for the editor failings in the methods (for example, in the experimental design and statistical analysis) or reasoning (for example, interpreting the results within the context of previous knowledge). However, because of their diverse cultural backgrounds, not all reviewers and editors will have the same expectations for argumentation and internal coherence.

### When gatekeepers and writing professionals work together

More than 10 years ago Richard Horton reflected on the suggestion that peer review was the equivalent of nothing more than good technical editing. Horton understood that peer review processes take place within two spheres: subject expertise and language expertise. Missing from peer review, he maintained, was the ability to provide authors with feedback on how persuasive their arguments were. He suggested that critical review of manuscripts by linguists could determine how effectively the authors had used language to support their point of view. "Such an analysis is part of the critical culture of science and would be a very welcome third component of peer review, in addition to qualitative and statistical assessment." [25] The reason why no journals seem to have acted upon Horton's suggestion to add rhetorical review to their peer review process may be related to editors' and reviewers' understandable lack of skill in the specialized task of applying "textual criticism of scientific discourse" to judge how persuasive a manuscript is. Such analyses are the domain of applied linguistics and discourse analysis, and require specialized knowledge to perform competently.

However, a few bold medical journal editors have ventured to work with experts in applied linguists to investigate the challenges authors face when they try to write their research articles well in English. Thoracic surgeon and editor John R. Benfield, working with linguist Christine B. Feak, suggested that authors who use English as an international language need input from both language professionals and experienced peers [26]. This view–that two separate skill sets are involved in providing useful feedback that will help researchers become proficient, successful writers–echoes the evidence from research in language and writing [24,27-31]. Benfield had become convinced that "peers and language professionals working together are more effective as editors" than either type of corrector alone in improving research articles written by authors whose first language is not English [32].

At the *Croatian Medical Journal *gatekeeper editors together with a manuscript editor analyzed how peer review could be used to teach researchers how to write well [33]. These editors perceived a need to provide intensive support to authors because they recognized that researchers often had valuable hypotheses and data but lacked the skills to present them. This led the editors to develop "an instructional editorial policy to increase the critical mass of researchers competent in scientific writing." As a result, the editors of *Croatian Medical Journal *developed author-helpful interventions to improve writers' competencies in four dimensions: study design, narrative, scientific reporting style and language.

These editors observed that translators used by the authors in their setting (a small central European country) often had "insufficient knowledge of medicine and the rules of scientific writing," but nonetheless believed that "the translator or language professional aware of [the] deep intellectual and informational need behind every recommendation within the ICMJE recommendations could substantially contribute to the quality of the manuscript by correcting or pointing out drawbacks (content-, structure- or language-related) of the manuscript to authors before they submit it for publication" (p. 130). This type of editorial input is in fact exactly within the remit of author's editors and "translators as editors" who work with researchers [34-38]. Wordface experts are already offering workshops to train non-subject-specialist language and writing professionals to handle specialist material competently [39,40].

Editors at *Annals of Emergency Medicine *have defined the two main functions of peer review in these words, " [w]e perform peer review not merely to select the best science but to improve it before publication." [41] Accordingly, this journal recommends that authors use "clear, succinct prose" and that they consider research reports as a "story," i.e., "an attempt to communicate an experience" that "brings the reader as close to the actual experience as possible." Its instructions to authors emphasize that manuscripts should be written in "the most direct" and "the clearest" manner possible. But the editors' criteria for clarity, succinctness or directness are not made specific. Readers' perceptions of these features may vary considerably, and may not be shared by all the journal's reviewers.

To clarify what this journal expects its peer review process to achieve, it made public its criteria for rating review quality [42] and subsequently explained these criteria more fully in the journal's Guide for Reviewers [43]. Two of the six criteria this journal uses to evaluate the quality of the reviews show an awareness that writing quality should be considered separately from scientific quality (from Table 1 in reference 42):

The reviewer commented upon major strengths and weaknesses of the manuscript as a written communication, independent of the design, methodology, results, and interpretation of the study.

The reviewer provided the author with useful suggestions for improvement of the manuscript. ("improvement of the manuscript" could refer to the content or the language/writing, or to both).

It will be interesting to see how useful the explicit distinction between content and writing has been in helping reviewers to provide more useful feedback to authors.

## Conclusions: How can gatekeepers make peer review better at improving the language and writing?

For editors who feel their journal's peer review process is due for critical review, the first document to scrutinize is the guidelines to authors. Although most journals provide many detailed instructions about style, usage and formatting, they offer little advice about how to write effective text [18]. As an aid to authors who hope to satisfy gatekeepers' expectations for good writing, it is helpful to explain the criteria reviewers use to evaluate manuscripts, especially if criteria other than the quality of the scientific content are used [23,44].

Another possible target for review is the set of guidelines for reviewers. Differentiating clearly between content-based and writing-based criteria may help reviewers focus on the parts of manuscripts they are most competent to judge. Offering guidance on how to provide useful feedback and when to withhold feedback may improve the usefulness of reviewers' reports to authors. Encouraging objectivity and a degree of flexibility regarding "good scientific English style" may reduce the amount of unhelpful feedback about language and writing. If reviewers are asked to advise authors on how to improve the writing, reviewers need better guidance on how to do this successfully. If reviewers feel uncertain about their ability to offer helpful feedback on the use of English or the quality of the writing, they should refrain from criticizing these features.

Academic writing for publication can adopt many rhetorical structures and styles, and not all reviewers or editors will be skilled in unpacking the information from all variants. Every author wants a respectful reading, [1] and although a particular piece of writing may not meet all a given reader's expectations–at least not initially–reviewers who try to read more respectfully may discover new keys to understanding that enable them to provide more constructive feedback than an unhelpful blanket complaint about "the English."

How can editors test what types of writing and editing make published articles more comprehensible, readable and useful to readers? The only way is to ask a representative sample of real readers to rate characteristics of the text [4,5]. Designing such research would probably require consultation with experts in academic literacy and other specialists in writing and editing. Fortunately, such experts are available [45,46], and working with them might help overcome some of the obstacles to peer review research noted by Callaham and Tercier [6] when they concluded,

[...] reviewer performance may be based on qualities for which we have not as yet determined good methods of identification and measurement, such as skepticism, thoroughness, motivation, inherent talent in detecting design weaknesses, etc. Skill in scientific peer review may be as ill defined and hard to impart as is "common sense," particularly if reviewers' decision-making is based on intuitive recognition of complex patterns of "quality" in the manuscript and not on rational analysis of simple components."

Researchers can turn to three potential sources of information to help make peer review a more reliable, constructive process. Gatekeepers can provide advice on models of peer review and reviewer evaluation strategies that have been found effective. Wordface professionals such as authors' editors and translators can provide insights into the types of feedback authors find most useful. Academic literacy researchers can identify features of good writing that are likely to make published articles more successful with readers. All three groups share the goal of helping "international" authors on the periphery of their discourse communities [47] to participate in conversations about science taking place in respected specialized journals and at prestigious conferences.

The greatest understatement regarding journal quality control is probably, "the methodological problems in studying peer review are many and complex." [48] Sharing expertise in the research methods and knowledge about English native-speaking and non-native-speaking authors' research culture might help editologists to design better studies and obtain results that can be applied to real-life writing, revising, peer review and editing. If a "large, well-funded programme of research on the effects of editorial peer review" is ever launched [48] it would be useful for gatekeepers who wish to publish better-written, more persuasive and more easily understood research articles to seek input from the other two communities of experts in scientific, technical and medical communication.

## Summary

1. For editors who wish to improve peer review processes, it may be useful to examine the research and methods used in disciplines outside the gatekeeper's own specialty for ideas on how to refocus their own research.

2. It may be useful to examine findings reported by nonacademic communication professionals for insights into what authors and readers would like peer review to accomplish.

3. For editors who expect peer review to provide effective feedback about language and writing, it would be useful to learn about research in academic writing for an international readership.

4. Nothing about the effectiveness of a text as a written communication can be known for certain unless real target readers are asked to judge the quality of the texts. Editorial interventions that have been "tested in real readers" should be considered a marker of editorial quality.

5. Gatekeepers, academic literacy researchers and wordface professionals such as author's editors, medical writers and translators could work together to identify the types of feedback authors find most useful in helping them bring their manuscripts up to publication standards.

## Abbreviations

AMA, American Medical Association; STM, scientific, technical and medical

## Competing interests

The author is a freelance translator, author's editor and editorial consultant. Publication of this article might attract clients to her business.

## Authors' contributions

KS conceived the study, carried out the literature review and analysis, collected and analyzed the data, drafted the manuscript, and was responsible for all subsequent drafts and the final version.

## Pre-publication history

The pre-publication history for this paper can be accessed here:



## Supplementary Material

Additional File 1 Publishers' language policies. Additional text and references Click here for file

Additional File 2 Americal Psychological Association and American Chemical Society language policies. Additional text and references Click here for file

Additional File 3 Test of the 2-category coding system. Additional text, references, and tables Click here for file

Additional File 4 Academic research. Additional text and references Click here for file

Additional File 5 Wordface research. Additional text and references Click here for file
