# Supplementary material for: Content and communication: How can peer review provide helpful feedback about the writing?
Source: BMC Med Res Methodol. 2008 Jan 31;8:3. doi: 10.1186/1471-2288-8-3 (PMC2268697; doi:10.1186/1471-2288-8-3)
Supplement: Additional File 1 — Publishers' language policies. Additional text and references [file 1471-2288-8-3-S1.doc]

**Shashok Debate - Additional File 1**

*Publishers’ language policies*

A comparison of the instructions for manuscript preparation across major science, technical and medical (STM) publishers reveals more evidence of decreasing tolerance for less-than-perfect language and writing. The instructions to authors of the *European Journal of Epidemiology,* published by Springer, represent the author-friendly end of the scale: “We appreciate any efforts that you make to ensure that the language is corrected before submission. This will greatly improve the legibility of your paper if English is not your first language.” [1] For other Springer journals, however, the instructions for authors mention only that manuscripts should be written in English, but do not require authors to take extra measures to ensure their manuscripts will need as little editing as possible. Springer’s information under the link to “Preparing a Journal Manuscript” refers authors to the homepage of the specific journal the author is interested in, [2] so this publisher appears not to apply a blanket policy to all its journals, but to leave editorial policy on the quality of English at the discretion of individual journal editors.

Blackwell (now Wiley-Blackwell) is a little more proactive in suggesting that “[a]uthors for whom English is a second language may choose to have their manuscript professionally edited before submission or during the review process.” [3]

At Wiley [4] the message for authors is stern: get the manuscript edited professionally before submittal or risk rejection. This publisher states that they will not undertake language editing:

We recognize that many manuscripts that are submitted to our journals originate from outside English-speaking countries. We strongly recommend that authors who are not native English speakers ask a colleague or for-hire editor whose native language is English to help them edit their work. Neither the editorial office nor the publishing staff are able to provide this service, and if papers are not written in easily understandable, idiomatic English, they will likely be returned to the authors without review.

According to Elsevier, “[e]ditors and reviewers are not responsible for making language corrections.” Even though the manuscript may report cutting-edge science, “poor language quality –including errors in grammar, spelling or language usage– could delay publication or could lead to outright rejection of the paper, preventing the research from getting the recognition it deserves.” [5] This policy not only implies that poor English may bias peer reviewers against the manuscript, but makes clear that Elsevier no longer considers editing the English as the publisher’s responsibility.

A factor that contributes to decreasing editorial tolerance may be the need to reduce costs, especially at open access journals. For example, in the instructions to authors for *BMC Medical Research Methodology*, [6] the advice on “Style and language” contains this information:

*BMC Medical Research Methodology* will not edit submitted manuscripts for style or language; reviewers may advise rejection of a manuscript if it is compromised by grammatical errors. Authors are advised to write clearly and simply, and to have their article checked by colleagues before submission. In-house copyediting will be minimal. Non-native speakers of English may choose to make use of a copyediting service.

Except for its two flagship journals *BMC Biology* and *BMC Medicine* (Matthew Hodgkinson, personal communication, e-mail 6 October 2007), this publisher declines responsibility for editing the English, and warns that manuscripts that are “compromised” by errors risk rejection without review.

**References**

1. Anonymous: **Springer, European Journal of Epidemiology, Instructions for Authors.** 2007. [http://www.springer.com/cda/content/document/cda_downloaddocument/instr_print_10654.070426.pdf?SGWID=0-0-45-127347-p35710057]. Accessed 14 August 2007.

2. Springer. **For Authors. Preparing a Journal Manuscript.** 2007. [http://www.springer.com/west/home/authors/manuscript+guidelines?SGWID=4-40162-12-357799-0&teaserId=329394&CENTER_ID=351199]. Accessed 24 October 2007.

3. Anonymous: **Blackwell Publishing, Author services, Pre-acceptance English-language editing.** 2007.

[http://www.blackwellpublishing.com/bauthor/english_language.asp] Accessed 14 August 2007.

4. Anonymous: **Wiley Interscience, Author Resources, Journal Manuscript Submission Guidelines, Language editing.** 2007. [http://www3.interscience.wiley.com/authorresources/journal-man-sub.html#langed]. Accessed 15 August 2007.

5. Anonymous: **Elsevier. Language Editing & Quality.** 2007. [http://www.elsevier.com/wps/find/authorsview.authors/languageediting/languageediting]. Accessed 14 August 2007.

6. Anonymous: **Instructions for BMC Medical Research Methodology authors.** 2007. [http://www.biomedcentral.com/bmcmedresmethodol/ifora/]. Accessed 24 July 2007.
