# Supplementary material for: Content and communication: How can peer review provide helpful feedback about the writing?
Source: BMC Med Res Methodol. 2008 Jan 31;8:3. doi: 10.1186/1471-2288-8-3 (PMC2268697; doi:10.1186/1471-2288-8-3)
Supplement: Additional File 2 — Americal Psychological Association and American Chemical Society language policies. Additional text and references [file 1471-2288-8-3-S2.doc]

**Shashok Debate - Additional File 2**

*Americal Psychological Association and American Chemical Society language policies*

The Publication Manual of the American Psychological Association (APA), a style manual used as a de facto standard by many psychology and social science journals, [1] devotes a whole chapter to writing and style (Chapter 2, “Expressing ideas and reducing bias in language”), and advises on writing style, grammar, and avoiding bias in language. However, the compliance gatekeepers perceive with the recommendations in the “Writing style” section for orderly presentation of ideas, smoothness of expression, economy of expression, precision and clarity, and strategies to improve writing style may depend on subjective judgments rather than on evidence that certain writing strategies are more effective than others.

The APA Publication Manual distinguishes clearly between writing and style guidelines. Chapter 3, on “APA editorial style,” provides prescriptive instructions on punctuation, spelling, capitalization, italics, abbreviations, headings and series, quotations, citation, and reference formats—aspects that used to be the domain of copyeditors but that researchers in psychology are now expected to get right in manuscript stage.

The American Chemical Society’s (ACS) style manual [2] offers suggestions on how authors can create an environment favorable to productive writing, and warns that “clutter” is often a problem in “American writing.” It also offers advice to authors who are preparing to write: decide on the function or purpose of the paper, identify what is novel about your contribution, chose the best journal, and decide who the audience is. However, the rest of the advice about “writing style” centers on usage of words and phrases rather than on how writers can produce a manuscript that tells an engaging, persuasive story about their research.

More revealing information on what reviewers and editors believe to be the functions of peer review is found elsewhere in the ACS manual, in Chapter 10. Gatekeepers were asked for their opinions on what peer review is and why it is done, and this chapter provides a wealth of data on what reviewers in this discipline believe they should do, and what kinds of feedback they expect themselves.

An analysis of their responses reveals a wide variety of opinions and some interesting contradictions. About 60% of the respondents felt it was part of a reviewer’s job to offer feedback on the writing, but some noted that this function was “not central,” “of less value,” or “secondary,” and some said that they did not feel they should be expected to edit the manuscript or improve the English in submittals from “foreign authors.” When responding as authors themselves, however, about 60% of the gatekeepers did not include this type of feedback among their expectations.

**References**

1. American Psychological Association: *Publication Manual of the American Psychological Association*. 5th Edition. Washington, D.C.: American Psychological Association; 2001.

2. Dodd JS (Ed): *The ACS Style Guide. A Manual for Authors and Editors.* 2nd edition. Washington, D.C.: Americal Chemical Society; 1997.
