# Supplementary material for: Content and communication: How can peer review provide helpful feedback about the writing?
Source: BMC Med Res Methodol. 2008 Jan 31;8:3. doi: 10.1186/1471-2288-8-3 (PMC2268697; doi:10.1186/1471-2288-8-3)
Supplement: Additional File 3 — Test of the 2-category coding system. Additional text, references, and tables [file 1471-2288-8-3-S3.doc]

**Shashok Debate - Additional File 3**

*Test of the 2-category coding system*

I looked at a sample of reviewer forms for seven journals and tried to classify the criteria reviewers are asked to consider. I was able to assign most criteria to one category or the other. The one criterion out of 62 that I could not assign asked reviewers to judge who in the academic medicine community would be interested in reading the paper, or should read the paper.

**Table 1. Classification of criteria in 7 medical journals’ instructions to reviewers [from reference 1]**

| Classification as pertaining to content or writing | Number | Percentage |
| --- | --- | --- |
| Total number of criteria classified | 62 |  |
| Range in number of criteria across journals | 3 – 24 |  |
| Mean number of criteria for all 7 journals | 8.8 |  |
| Total number of criteria classified as content | 44 | 71.0% |
| Mean percentage of criteria classified as content |  | 71.0% |
| Total number of criteria classified as writing | 17 | 27.4% |
| Mean percentage of criteria classified as writing |  | 26.9% |
| Total. number of unclassifiable criteria: | 1 | 1.6% |

I also tested the 2-category system by coding the feedback in a recent set of reviewers’ reports as pertaining to content or writing, and was able to assign most comments to one category or the other.

**Table 2. Classification of feedback in two reviewers’ reports on a manuscript submitted to an epidemiology journal**

| Classification as pertaining to content or writing | Number | Percentage |
| --- | --- | --- |
| Total number of comments classified | 91 |  |
| Number of comments classified as content | 61 | 67.0% |
| Number of comments classified as writing | 27 | 29.7% |
| Number of unclassifiable comments | 3 | 3.3% |

Reviewer 1 provided 79 comments; Reviewer 2 provided 12 comments. Reviewer 1 offered twice as many comments about the content (n = 54) as about the writing (n = 22); Reviewer 2 provided only slightly more comments about the content (n = 7) than about the writing (n = 5).

**Reference**

1. Joint Task Force of Academic Medicine and the GEA—RIME Committee: **Review criteria for research manuscripts. (Appendix 3: Sample review forms.)** *Academic Medicine* 2001; 76(9): 897-978.
