# Supplementary material for: Content and communication: How can peer review provide helpful feedback about the writing?
Source: BMC Med Res Methodol. 2008 Jan 31;8:3. doi: 10.1186/1471-2288-8-3 (PMC2268697; doi:10.1186/1471-2288-8-3)
Supplement: Additional File 4 — Academic research. Additional text and references [file 1471-2288-8-3-S4.doc]

**Shashok Debate - Additional File 4**

*Academic research*

Relevant to the difficulties reviewers have in providing constructive feedback about the writing is the realization by academic literacy researchers Theresa Lillis and Mary Jane Curry that readers note problems with “the language” when in fact they may be referring to problems with any level of writing, from the most superficial (grammatical accuracy) to the deepest (proficient use of specialist discourse). [1]

First, although participants distinguish between types of brokers (friends, academics, translators), they tend to frame all their different interventions as relating to language, whether this is grammatical accuracy or specialist discourse (p. 16).

An important finding is that a large amount of brokering is carried out by academic professionals and that although scholars tend to frame these brokers’ interventions in terms of language or discourse, in fact they tend to orient to content. This raises important questions about the precise nature of the activities both groups of scholars assume they are taking part in (and why) (p. 29).

In other words, when reviewers and editors complain about “the English,” the problem may often lie not in spelling, grammar or syntax errors, but in the organization, order of information, flow of ideas, coherence, persuasiveness of the argumentation, or other elements of rhetoric—or even with deficiencies in the discipline-specific content.

Lillis and Curry have also examined how multilingual academics with English as an additional language made use of locally available writing help. [1] [2] Persons potentially able to help researchers improve their writing—called “literacy brokers” by Lillis and Curry—may be disciplinary specialists (the authors’ academic peers), journal gatekeepers, language specialists or Anglophone friends and relatives with no particular background in linguistics or academic discourse. Lillis and Curry cautioned that peer reviewers sometimes made changes in manuscripts that changed the original meaning, and that authors, although not always happy with these changes, were willing to “make sacrifices” if it meant that the manuscript would be accepted and published.

In their interpretation of the factors that affect text revision, Lillis and Curry put their finger on some of the questions peer review research by gatekeepers has so far failed to consider: What dimensions of a written communication *should* reviewers be expected to critique? What dimensions to reviewers *assume* they are expected to critique? What are reviewers actually *able* to critique competently—the language as well as the content, or only the latter?

Hugh Gosden, a specialist in foreign language research and teaching, has investigated ways to “elucidate for novice NNSE (non-native speakers of English) researchers how to interpret the ‘helpfulness’ of referee’s remarks in the task of revising their papers.” He notes that “[...] as referees’ criticisms become more general, reflecting greater perceived weaknesses in the overall manuscript and/or research itself, the more fundamental revisions would need to be, and the more difficult it would likely be for authors to extract help from referees’ comments.” [3] Although his analysis is based on the assumption that all the referees’ comments reflected “true positives,” i.e., actual problems in the manuscript (referees are sometimes wrong), this carefully documented descriptive analysis of case studies provides information that editors could apply to find out how useful their own reviewers’ comments are to authors.

Gosden’s research suggests that to be helpful to the author, reviewers’ comments about the writing should refer to the specific reason why the text has given the reviewer problems. In practical terms, this means is that instead of general comments such as “The text here is confusing” or “I don’t understand what the authors are driving at,” reviewers should tell authors “This sentence seems out of place here” or better still, “I don’t understand how this information relates to the information in the preceding (or following) text.” In other words, they should try to identify for the writer what malfunction in the text triggered their comprehension problem. The text, after all, may be quite clear to readers other than the reviewer. [4]

John M. Swales, a linguist who identified key functions of the different sections of research articles [5] structured with the IMRaD format (*i*ntroduction, *m*ethods, *r*esults *a*nd *d*iscussion), called reviewer’s reports and editorial correspondence between editors and authors “occluded genres” [6] which remain hidden from sight (and, until recently, mostly sequestered from academic analysis) because of the confidential nature of peer review. Now, fortunately, some journals have begun to open up their files to researchers. At the Publishing and Presenting Research Internationally: Issues for Speakers of English as an Additional Language (PPRISEAL) conference in January, 2007, [7] Inmaculada Fortanet, a Spanish researcher in academic writing, found that feedback about the writing provided by reviewers and editors was less helpful to authors than it might be. Her study [8] of reviewers’ reports in the disciplines of linguistics and business and management found that about 85% of the feedback consisted of complaints about what was wrong with the manuscript, with only 15% containing constructive advice. Spanish researchers in her study identified several difficulties with reviewer’s reports that made it harder than necessary for the authors to successfully revise the text. These difficulties were 1) criticisms not supported by reasoning (and therefore unlikely to convince authors that any change in the manuscript was needed), 2) reports that were so short that authors could not understand what was being asked of them, and 3) ambiguous comments that authors could not interpret with confidence.

Participants at the PPRISEAL conference expressed concern that overdependence on rhetorical systems that characterize English language writing might contribute to intolerance toward alternative patterns in language and discourse that arise from other languages. [9] Suresh Canagarajah, editor of the highly regarded journal *TESOL Quarterly*, [10] explained that English written by authors with a different first language can reflect their ability to shuttle between different writing strategies and meld elements of text in ways writers who know only English are unable to achieve. Texts produced by this “code-meshing” process can be just as effective as texts that follow the well-know sequence of rhetorical moves described by Swales’ system [5] of CARS analysis (*c*reating *a* *r*esearch *s*pace). Canagarajah urged editors and reviewers to be more flexible in judging the texts they receive for review, since writing strategies that depart from the “classical” sequence of rhetorical moves in experimental and health science articles can be used intentionally by authors to support arguments that the CARS structure is unable to make convincingly. Belcher [11] also challenged gatekeepers’ criteria for judging the quality of writing by asking whether Anglo-American conventions should “continue to be the unquestioned norm if contributions from around the world are sought and a truly global audience the goal of ‘international’ journals” (p. 19).

Interestingly, the constraints of the IMRaD format as a tool for written communication were also recently noted by Elizabeth Wager, a medical writer, editor and trainer. She observed that although the IMRaD format “has evolved as a useful workhorse,” it “may not be the best way to present findings to every audience.” Wager urged researchers to test different reporting methods on their target audiences to search for evidence of approaches to writing able to improve the efficacy of communication. [12]

**References**

1. Lillis T, Curry MJ: **Professional academic writing by multilingual scholars. Interactions with literacy brokers in the production of English-medium texts.** *Written Communication* 2006; 23(1) (January): 3-35.

2. Lillis TM, Curry MJ: **Reframing notions of competence in scholarly writing: From individual to networked activity.** *Revista Canaria de Estudios Ingleses* 2006; 53: 63-78.

3. Gosden H: **‘Why not give us the full story?’: functions of referees’ comments in peer review of scientific research papers.** *Journal of English for Academic Purposes* 2003; 2(2): 2-16.

4. Shashok K: **Speaking virtually the same language: coding and decoding messages between authors and editors.** *European Science Editing* 1994; 53 (Sept): 5-7.

5. Swales J: *Genre analysis: English in academic and research settings.* Cambridge: Cambridge University Press; 1990.

6. Swales, J: **Occluded genres in the academy: the case of the submission letter**. In *Academic Writing: Intercultural and Textual Issues.* Edited by Ventola E, Mauranen A. Amsterdam: John Benjamins; 1996: 45-58.

7. **Publishing and Presenting Research Internationally. Issues for Speakers of English as an Additional Language (PPRISEAL).** La Laguna, Tenerife, Canary Islands (Spain), 11-13 January 2007. [http://webpages.ull.es/users/ppriseal/].

8. Fortanet I: **Strategies for teaching and learning an occluded genre: the research article referee report.** In *English As an Additional Language in Research Publication and Communication (Linguistic Insights series).* Edited by Burgess S, Martín P. Bern: Peter Lang; 2008 (In press).

9. Shashok K: **Successful communication in English for non-native users of the language. International conferences make Spain a world leader in writing, editing and translation research.** *Panace@* 2007a; VIII (25): 82-86.

[http://www.medtrad.org/panacea/IndiceGeneral/n25_congresos-shashok.pdf.]

10. Anonymous: **About TESOL Quarterly.** 2007. [http://www.tesol.org/s_tesol/seccss.asp?CID=209&DID=1679]. Accessed 23 January 2007.

11. Belcher DD: **Seeking acceptance in an English-only research world.** *Journal of Second Language Writing* 2007; 16: 1-22.

12. Wager E: **What medical writing means to me.** *Mens Sana Monographs* 2007; 5: 169-178.

[http://www.msmonographs.org/article.asp?issn=0973-1229;year=2007;volume=5;issue=1;spage=169;epage=178;aulast=Wager;type=0]. Accessed 14 July 2007.
