# Supplementary material for: Content and communication: How can peer review provide helpful feedback about the writing?
Source: BMC Med Res Methodol. 2008 Jan 31;8:3. doi: 10.1186/1471-2288-8-3 (PMC2268697; doi:10.1186/1471-2288-8-3)
Supplement: Additional File 5 — Wordface research. Additional text and references [file 1471-2288-8-3-S5.doc]

**Shashok Debate - Additional File 5**

*Wordface research*

In experiment designed to ascertain what aspects of Dutch scientific English were noticed by peer readers, Burrough-Boenisch asked a sample of volunteer readers (who were the authors’ scientific peers, but who had different native languages) to annotate excerpts from the discussion section of manuscripts. [1] Her findings showed that the corrections these readers marked in the texts varied widely, and that it was difficult to find consistent patterns in the changes made—which were not always improvements. The readers were sometimes able to fix “superficial” problems with the language (e.g., spelling, grammar, and errors in word choice) but did not identify or correct deeper problems with the text (e.g., the intended focus of the sentence, logical connections between ideas, and rhetorical problems involving larger segments of the text). This led her to conclude that reviewers should not be used as language revisers: although their specialist knowledge enables them to improve the technical language, they do not necessarily notice first-language interference (i.e., where the English “sounds like” or “reads like” some other language) if they are not themselves familiar with the author’s first language.

Burrough-Boenisch subsequently published a description of the different functions of authors, authors’ editors, reviewers, editors and copyeditors in shaping the final text. [2] Her overview of different inputs into academic texts contains much information that may help editors and other peer review researchers understand the writing, review and editing processes. Notably, the section headed “Reviewers’ interventions” reports some interesting differences in reviewers’ performances and preferences in relation to their cultural background—differences editors of international journals may wish to take note of.

As readers, subject experts (in contrast to writing experts) focus on the content of texts, whereas authors’ editors [2] focus more on “rhetorical appropriateness” and “linguistic aspects.” Burrough-Boenisch noted that “reviewers for science journals are unlikely to be as linguistically alert and gifted as author’s editors, and, given their brief to assess the research article’s scientific merit, are less preoccupied with the linguistic aspects of the text” (p. 234).

In her research to discover factors that influenced how reviewers and non-reviewers from different cultural backgrounds judged Dutch-authored scientific texts in English, Burrough-Boenisch [1] found that “two interesting groups emerge: language professionals who are not subject (i.e., content) experts, and scientific peers, who may or may not be [native speakers of English]” (p. 23). Her findings “suggest that language revisers and scientists have different expectations from the English of a scientific text” (p. 122). This research supports the notion that advice on “the writing” offered by scientific peers may be less helpful to authors than advice offered by professional editors or other communication professionals.

**References**

1. Burrough-Boenisch J: **Culture and conventions: writing and reading Dutch scientific English.** Utrecht (The Netherlands): LOT Netherlands Graduate School of Linguistics (dissertation no. 59); 2002.

[http://www.lotpublications.nl/index.html]. Accessed 29 January 2007.

2. Burrough-Boenisch J: **Shapers of published NNS research articles.** *Journal of Second Language Writing* 2003; 12: 223-243.
